# Synthesis, Photophysical Properties, and Primary Biological Characteristics of Meso-pyrazole-substituted BODIPY Derivatives

**DOI:** 10.1007/s10895-026-04852-y

**Published:** 2026-06-24

**Authors:** Zehra Coşkun, Ercan Coşar, Burcu Topaloğlu Aksoy, Hasan Hüseyin Kazan, Ibrahim F. Sengul, Bünyemin Çoşut

**Affiliations:** 1 Department of Chemistry, Gebze Technical University, Gebze, 41400 Türkiye; 2 Department of Medical Biology, Gulhane Faculty of Medicine, University of Health Sciences, Ankara, Türkiye

**Keywords:** Photophysical Properties, BODIPY, Pyrazole, Bioimaging, Cellular toxicity

## Abstract

In the present study, two novel meso-pyrazole-substituted BODIPY platforms were synthesized by an acid-catalysed condensation reaction of pyrazole-4-carbaldehydes with 2,4-dimethylpyrrole, followed by complexation with BF_3_·OEt₂. Comprehensive investigations were carried out to characterize the structural, photophysical, and biological properties of the target BODIPY compounds. These investigations included structural elucidation through mass spectrometry and NMR spectroscopy, as well as spectroscopic analyses such as UV-Vis absorption, fluorescence emission, and quantum yield measurements. Compounds exhibited very similar absorption and fluorescence behaviors, with only minor shifts in their absorption and fluorescence maxima, indicating that minor structural differences had little influence on the overall photophysical properties. However, the fluorescence quantum yields of compounds 3 and 4 were found to be 0.82 and 0.33, respectively, despite their very similar absorption and emission characteristics. Additionally, primary biological characterizations were performed particularly regarding the biological importance of the coumarin unit. Accordingly, both novel compounds exhibited cytotoxicity against the human colorectal cancer cell line, with an inhibitory concentration 50 (IC50) value of 47.46 ± 3.69 and 83.56 ± 2.40 mg/ml. Moreover, the toxicities were proved to be a result of whether apoptosis or necrosis. Furthermore, the compounds were able to internalize into the cells, enabling live cell imaging. Notably, the coumarin-containing compound displayed aggregation-dependent alterations in photophysical properties with dual fluorescence, confirming the previous reports. Hence, developing such compounds is a feasible approach to comprehensively elucidate the photochemistry of BODIPY modulations and propose new application areas.

**Supplementary Information:**

The online version contains supplementary material available at 10.1007/s10895-026-04852-y.

## Introduction

Over the past decade, there has been growing interest in the development of BODIPY derivatives owing to their outstanding photophysical and photochemical properties, along with a range of biological activities [1]. BODIPY dyes are notable for their uniquely small Stokes shift, high fluorescent quantum yield, large molar absorption coefficient, good solubility in various organic solvents, and excellent chemical and photo stability [2–5]. These remarkable characteristics have made BODIPYs popular in a wide range of applications such as biochemical labeling, light-emitting devices, supramolecular fluorescent gels, light-harvesting systems, optical chemo-sensors, organic-thin film transistors, and sensitizers in solar cells [6–11].

BODIPY and its derivatives have proven to be extremely useful materials for biological studies [12, 13]. For instance, they can be employed as photosensitizers in photodynamic therapy applications due to their singlet oxygen capacity [12]. Another important application of BODIPY ring-based molecules is that these compounds can be used as fluorescence-based bioimaging agents [14]. It is worth noting that the BODIPY skeleton can easily be functionalized at all reactive sites, and such modifications provide an opportunity to fine-tune the electronic and photophysical properties [15]. Modifications at the meso position are especially important because this position is close to the fluorescent core, enabling modulation of BODIPY’s photophysical properties [16]. Consequently, fluorescence properties of BODIPY can be modulated by incorporating different substitution patterns at the meso position [16–19]. To date, numerous BODIPY derivatives have been modified at this position with different groups for bioimaging studies. For instance, Wu et al. recently synthesized a range of meso-*N*-acyl-BODIPYs, which showed bathochromic shifts, improved water solubility, larger Stokes shifts, and emission over a wide range. Their synthesized compounds were successfully used in live-cell imaging [16]. In another experiment, several meso-substituted thienyl BODIPYs were prepared by Singh et al., and the synthesized BODIPYs demonstrated biocompatibility toward endothelial cells and significant cytotoxicity against cancer cells [18].

It has been revealed from the literature that meso positions have been widely functionalized with various heterocyclic units in order to modulate its photophysical and biological properties [20–22]. Among these, pyrazole is a heterocyclic compound of the azole group and is characterized by a 5-membered ring composed of three carbon and two adjacent nitrogen atoms in adjacent positions [23]. Several studies have investigated the combination of pyrazole and BODIPY units. For example, research by Kamkaew et al. recently reported the synthesis of pyrazole-based aza-BODIPY as a novel photothermal agent that demonstrated excellent fluorescence signals in the cancer cells [24]. In another study, a series of pyrazole appended quinoline-BODIPY was synthesized by Pandey et al., which displayed in vitro antiproliferative activity against the human cervical cancer cell line (HeLa) [20]. Moreover, Xie et al. reported BODIPY nanoparticles with an electron donor pyrazoline unit for ultrafast and long-term bioimaging studies. Their study found that pyrazoline-substituted BODIPY illustrated an excellent performance in the field of long-term fluorescence imaging in vivo and could provide over 10 days of tumor fluorescence tracking for U14 tumor-bearing mice [25]. In addition to pyrazole derivatives, coumarin represents another important class of heterocyclic compounds broadly employed in medicinal and fluorescence chemistry [26, 27]. For example, Li et al. recently reported the design and synthesis of coumarin-functionalized BODIPY as a promising bioimaging and photodynamic therapy agent owing to their favorable photophysical properties, high photostability, and efficient cancer cell targeting ability [28].

Despite the promising results in functionalized BODIPY platforms, the development of BODIPY derivatives incorporating pyrazole and coumarin units remain limited. In particular, phenyl-linked pyrazole frameworks are attractive structural units because they can influence the electronic communication and biological behavior of fluorophoric systems through extended conjugation. Therefore, the combination of phenyl-linked pyrazole and coumarin groups into the BODIPY framework may provide enhance advantages in terms of photophysical properties and cytotoxic potential.

Specifically, considering the coumarin unit, primary biological assessments including cellular toxicities, imaging and aggregation-dependent photophysical alterations in biological systems would further support understanding of the nature of pyrazole-linked BODIPY complexes. Based on this, in the present study, novel meso-substituted BODIPY derivatives containing phenyl-linked pyrazole and coumarin units were designed and synthesized, and their photophysical properties together with cytotoxic activities were systematically investigated for potential biomedical applications.

## Experimental Section

### Materials

All chemicals and starting materials for the synthesis part were provided by Sigma Aldrich, a commercial supplier, and they were used without extra purification. The solvents used in the synthesis process and spectroscopic analysis were procured from Merck. Reactions were controlled with thin-layer chromatography using Merck TLC Silica gel 60 F254. Silica gel column chromatography was carried out over Merck Silica gel 60 (particle size: 0.040–0.063 mm; 230–400 mesh ASTM).

### Equipment for Chemical Analysis

HRMS spectra of the synthesized compounds were recorded from methanol solutions using a Waters LCT Premier XE UPLC/MS-TOF system, equipped with an electrospray ionization (ESI+) source and managed via MassLynx 4.1 software. An Acquity BEH C18 column (2.1 × 100 mm, 1.7 μm) was employed as the stationary phase at a flow rate of 0.3 mL/min. The mobile phase consisted of a gradient system of CH_3_CN: H_2_O (ranging from 1% to 90%) containing 0.1% formic acid. For ^1^H and ^13^C-NMR measurements, spectra were recorded for all compounds in CDCl_3_ on a Varian INOVA 500 MHz spectrometer using TMS as an internal reference. For the photophysical characterization, electronic absorption spectra in the UV-Vis region were recorded with a Shimadzu 2101 UV-Vis spectrophotometer. Fluorescence measurements were carried out with a Varian Eclipse spectrofluorometer using 1 cm path length cuvettes at room temperature. The fluorescence lifetimes were obtained using a Horiba-Jobin-Yvon-SPEX Fluorolog 3-2iHR instrument with Fluoro Hub-B Single Photon Counting Controller for target compounds. For signal acquisition, a TCSPC module was chosen. Perkin Elmer Spectrum 100 model Attenuated Total Reflectance (ATR) Fourier Transform Infrared Spectroscopy was used to further characterize the synthesized compounds.

### Synthesis

#### Synthesis of Precursors

**Scheme 1:**
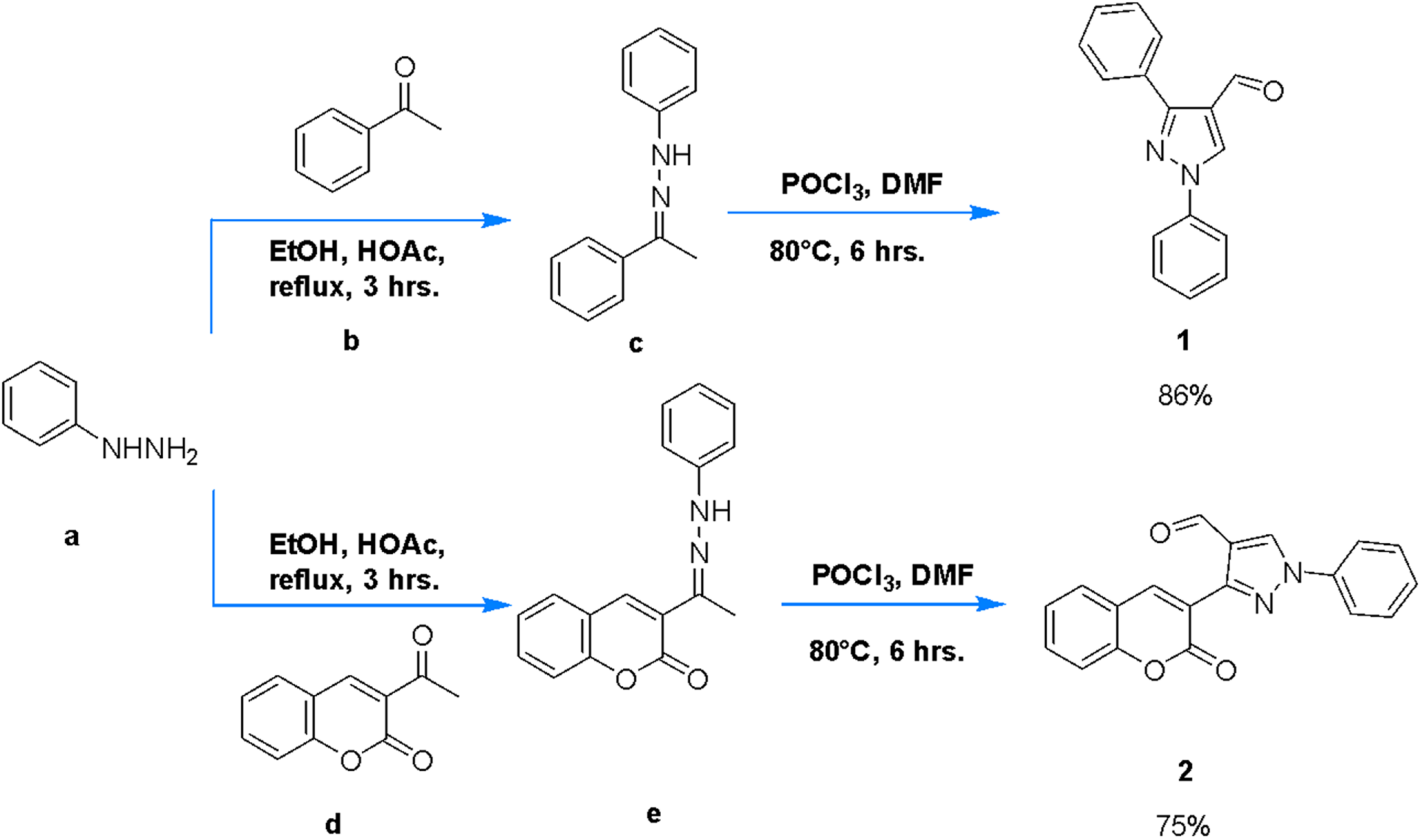
Reagents and synthesis route of the precursor compounds **1** and **2**

Precursor compounds (**1** and **2**) were prepared according to procedures described previously (Scheme 1) [29, 30]. Briefly, acetophenone was reacted with phenylhydrazine to generate the corresponding phenyl hydrazone intermediate, which then cyclized under Vilsmeier-Haack reaction conditions using POCl_3_ and DMF to yield compound **1** [26]. Similarly, the reaction of 3-acetylcoumarin with phenyl hydrazine under the same reaction conditions gave the corresponding coumarin-substituted pyrazole **2** in good yield [30].

##### ^1^H and ^13^C NMR of 1

Compound **1** was obtained as a white solid (86%): ^1^H NMR (500 MHz, CDCl_3_) δ 10.09 (s, 1H, CHO), 8.58 (s, 1H, ArH), 7.84 (dd, *J* = 12.6, 8.0 Hz, 4 H, ArH), 7.53 (dt, *J* = 7.2, 5.8 Hz, 5 H, ArH), 7.42 (t, *J* = 7.4 Hz, 1H, ArH) (Figure S1). ^13^C NMR (126 MHz, CDCl_3_) δ 185.3 (CHO), 154.9, 139.1, 131.4, 130.9, 129.7, 129.3, 129.0, 128.8, 128.0, 122.5, 119.8 (Figure S2).

##### ^1^H and ^13^C NMR of 2

Compound **2** was obtained as a yellow solid (75%): ^1^H NMR (500 MHz, CDCl_3_) δ 9.98 (s, 1H, CHO), 8.51 (s, 1H, ArH), 8.17 (s, 1H, ArH), 7.71 (d, *J* = 7.7 Hz, 2 H, ArH), 7.56 (d, *J* = 7.4 Hz, 2 H, ArH), 7.46 (t, *J* = 7.9 Hz, 2 H, ArH), 7.41–7.34 (m, 2 H, arH), 7.30 (d, *J* = 7.4 Hz, 1H, ArH) (Figure S3). ^13^C NMR (126 MHz, CDCl_3_) δ 185.1 (CHO), 160.1, 154.1, 147.7, 143.2, 138.9, 132.5, 131.1, 129.7, 128.6, 128.2, 124.9, 123.9, 120.1, 119.8, 119.0, 116.7 (Figure S4).

#### Synthesis of 3

A 500 mL round-bottomed flask was charged with dichloromethane (300 mL) and purged with argon (Ar) for 15 min. Then, 1,3-diphenyl-1H-pyrazole-4-carbaldehyde **1** (200 mg, 0.806 mmol) and 2,4-dimethylpyrrole (0.184 mL, 1.77 mmol) were added to the medium, respectively. After adding two drops of trifluoroacetic acid, the reaction was stirred at room temperature for 17 h. *p*-Chloranil (192 mg, 0.806 mmol) was added to the reaction mixture. The reaction mixture was stirred for 30 min, and triethylamine (6 mL) was added drop by drop and stirred for another 30 min. Lastly, BF_3_. OEt_2_ (6 mL) was added to the mixture drop by drop, and the reaction mixture was stirred at room temperature for an additional 3 h. The solvent was removed under reduced pressure. Compound **3** was isolated from column chromatography with silica gel (2:1; DCM: Hexane) yield: 90 mg (24%). HR-MS (m/z) [M]^+^: calculated for [C_28_H_25_BF_2_N_4_] ^+^ 466.214; found 466.231 (Figure S5). ^1^H NMR (500 MHz, CDCl_3_) δ 7.90 (d, *J* = 7.5 Hz, 2 H, ArH), 7.73 (d, *J* = 7.7 Hz, 2 H, ArH), 7.44 (dt, *J* = 8.9, 4.6 Hz, 3 H, ArH), 7.33 (dt, *J* = 14.9, 7.1 Hz, 2 H, ArH), 7.21 (d, *J* = 8.3 Hz, 1H, ArH), 7.16 (d, *J* = 7.0 Hz, 1H, ArH), 5.93 (s, 2 H, ArH), 2.47 (s, 6 H, 2xCH_3_), 1.68 (s, 6 H, 2xCH_3_) (Figure S6); ^13^C NMR (126 MHz, CDCl_3_) δ 157.6, 154.8, 152.8, 144.1, 141.6, 140.5, 138.3, 131.3, 131.1, 130.6, 128.7, 127.4, 126.5, 126.2, 123.4, 120.5, 119.3, 118.3, 117.9, 115.9, 115.5, 14.04 (CH_3_), 13.66 (CH_3_) (Figure S7); ^11^B NMR (160 MHz, CDCl_3_) δ − 0.98 (t) ppm (Figure S8); ^19^F NMR (471 MHz, CDCl_3_) δ − 150.8 (q) ppm. Figure S9) FT-IR: ʋmax 2969, 2925, 1728, 1601, 1546, 1468, 1408, 1366, 1241, 950, 836, 780, 751 cm^− 1^ (Figure S10).

#### Synthesis of 4

A 500 mL round-bottomed flask was charged with dichloromethane (250 mL), and purged with argon (Ar) for 15 min. Then, coumarin-substituted pyrazole-4-carbaldehyde **2** (200 mg, 0.635 mmol) and 2,4-dimethylpyrrole (0.145 mL, 1.4 mmol) were added to the medium, respectively. After adding two drops of trifluoroacetic acid, the reaction was stirred at room temperature for 17 h. *p*-Chloranil (151 mg, 0.635 mmol) was added to the reaction mixture. The reaction mixture was stirred for 30 min, and triethylamine (5 mL) was added drop by drop and stirred for another 30 min. Lastly, BF_3_. OEt_2_ (6 mL) was added to the mixture drop by drop, and the reaction mixture was stirred at room temperature for an additional 3 h. The solvent was removed under reduced pressure. Compound **4** was isolated from column chromatography with silica gel (1:99; MeOH: DCM) yield: 50 mg (15%). HR-MS (m/z) [M-F] ^+^: calculated for [C_32_H_28_BFN_4_O_2_] ^+^ 515,205; found: 515.016 (Figure S11). ^1^H NMR (500 MHz, CDCl_3_) δ 7.84 (s, 1H, ArH), 7.74 (d, *J* = 8.2 Hz, 4 H, ArH), 7.44 (t, *J* = 8.0 Hz, 2 H, ArH), 7.30–7.20 (m, 4 H, ArH), 5.91 (s, 2 H, ArH), 2.50 (s, 6 H, 2xCH_3_), 1.59 (s, 6 H, 2xCH_3_) (Figure S12); ^13^C NMR (126 MHz, CDCl_3_) δ 154.9, 148.5, 141.9, 138.5, 131.8, 131.2, 128.6, 128.0, 127.5, 127.2, 126.1, 125.4, 124.3, 120.4, 117.8, 113.8 (ArC), 13.81 (CH_3_) (Figure S13); ^11^B NMR (160 MHz, CDCl_3_) δ 0.77 (t, J = 32 Hz) (Figure S14); ^19^F NMR (471 MHz, CDCl_3_) δ − 146.2 (q, 32 Hz)0.150.8 (q) ppm. Figure S15); FT-IR:ʋmax 2993, 1636, 1473, 1312, 1030, 839, 715 cm^− 1^ (Figure S16).

### Fluorescence Quantum Yield Measurements

The fluorescence quantum yields of compounds were determined in DMSO by comparing to the fluorescence of Rhodamine 6G as a standard, respectively. Fluorescence quantum yields (Φ_F_) were calculated by the comparative method (Eq. 1) [31]

1 $${\varPhi\:}_{F}={\varPhi\:}_{F}\left(std\right)\frac{F.{A}_{std}\:{n}^{2}}{{F}_{std}.A.{n}_{std}^{2}}$$

where Φ_F_(Std) is the fluorescence quantum yield of the standard. Rhodamine 6G was employed as the standard for compounds (Φ_F_ = 0.95 in water) [32]. F and F_Std_ are the areas under the fluorescence emission curve of the sample and the standard, respectively. A and A_Std_ are the respective absorbance of the samples and standard at the excitation wavelengths. $\eta^2$ and $\eta_\mathrm{Std}^2$ are the refractive indices of solvents used for the sample and standard, respectively.

### X-ray Data Collection and Structure Refinement

Unit cell measurements and intensity data collection were performed on a Bruker APEX II QUAZAR three-circle diffractometer using monochromatized Mo Kα X-radiation (λ = 0.71073 Å). Indexing was performed using APEX2 [33]. Data integration and reduction were performed using SAINT V8.34 A [34]. Absorption correction was performed by the multi-scan method implemented in SADABS V2014/5 [35]. The structures were solved and refined using the Bruker SHELXTL Software Package [36]. All non-hydrogen atoms were refined anisotropically using all reflections with I > 2σ(I). The C-bound H atoms were positioned geometrically and refined using a riding mode. The N-bound H atoms were located from the difference Fourier map and and restrained to be 0.89 Å from N atom using DFIX and their position were constrained to refine on their parent N atoms with Uiso(H) = 1.2Ueq(N). The final geometrical calculations and the molecular drawings were carried out with Platon (version 1.17) and Mercury CSD (version 3.5.1) programs [37, 38]. CIFs were deposited with the Cambridge Crystallographic Data Centre (CCDC 2530328 for 3).

### Biological Studies

#### Cell Culture

The biological properties, including cytotoxicity and live cell imaging of the novel compounds (**3** and **4**) were investigated in the human colon cancer cell line, HCT-116. Cells were grown in Dulbecco’s modified Eagle’s medium (DMEM; Thermo Fisher Scientific, USA) containing 10% fetal bovine serum (FBS; Thermo Fisher, USA) and 1% penicillin-streptomycin (Thermo Fisher, USA) at 37 °C and 5% CO_2_. Before any treatment, cells were passaged at least two times. During the passaging, cells were collected by Trypsin (Sigma Aldrich, USA) and centrifugation, and diluted at proper amounts after washing with phosphate-buffered saline; (PBS; Sigma Aldrich, USA). All treatments were performed when the cells were at 80% confluency.

#### Cell Viability Assay

Toxicities of the compounds were evaluated using 3-(4, 5-dimethylthiazol-2-yl)−2,5-diphenyltetrazolium bromide (MTT) assay. Cells were seeded into the wells of 96-well plate at a concentration of 1 × 10^4^ cells/well and incubated overnight. Next, cells were washed by PBS twice, and the mediums containing diverse concentrations (0–100 mg/ml) of compounds dissolved in dimethyl sulfoxide (DMSO) were added onto the cells. The cells were further incubated for 48 h in the presence of the compounds. Then, 10 µl of 5 mg/ml MTT solution (Sigma Aldrich, USA) were added onto the cells and incubated for 4 h. Finally, cells were disrupted by 100 µl sodium dodecyl sulfate (SDS)-HCl solution (prepared by dissolving 1 g SDS in 10 ml molecular-grade water in the presence of 0.01 M HCl) by incubation for 24 h. Colorimetric alterations were followed by a plate spectrophotometer (Multiscan GO; Thermo Fisher Scientific, USA) at 570 nm. During the experiments, at least 3 technical replicas for each compound and concentration were used. Untreated, DMSO-only, and medium plus compounds only groups were used as controls. For analysis, the absorbances of the medium controls were subtracted from those of compound-treated cells. The viabilities of the results of the untreated group were assumed as 100%, and those of the treated groups were correlated with the untreated group. The inhibitory concentration 50 (IC_50_) values of the compounds were determined by a nonlinear regression test using GraphPad Prism 8.4.2 software (GraphPad Inc., USA).

#### Live Cell Imaging

Interaction and/or internalization of the compounds with/into the cells was followed by a live cell imaging method. Cells were seeded into the wells of a 6-well plate at a ratio of 2 × 10^5^ cells/well. After incubation for 24 h, cells were washed with PBS twice, treated with 1 mg/ml compound **3** or **4** in the presence of a solvent control group, and incubated for 5 min at room temperature. The cells were intentionally imaged without washing steps. For imaging, FLoid Cell Imaging Station (Thermo Fischer Scientific, USA) with green (excitation: 482/18 nm and emission: 532/59 nm) and red (excitation: 586/15 nm and emission: 646/68 nm) was used. The images were obtained from at least three different regions of the wells. After obtaining images without a washing step, the cells were washed with PBS twice and incubated with 5 µl of Trypan Blue solution (0.4%; Thermo Fisher Scientific, USA) in 1 ml PBS to block the extracellular fluorescence. Under these conditions, the imaging process was repeated.

#### Assessment of Cell Death

Considering the dual fluorescence nature of the compounds, fluorometric examinations could not be performed. Thus, the effect of compounds on cell death was evaluated using Trypan Blue staining. For Trypan Blue staining, cells in 6-well plates were exposed to 50 mg/ml concentrations of **3** or **4** in the presence of a DMSO control for 24 h. Next, cells were washed with PBS twice and treated with 0.4% Trypan Blue solution for 5 min at room temperature. After this duration, cells were washed with PBS twice and imaged under a light microscope for morphological and color-based investigations.

## Results and Discussion

### Synthesis and Characterization

**Scheme 2:**
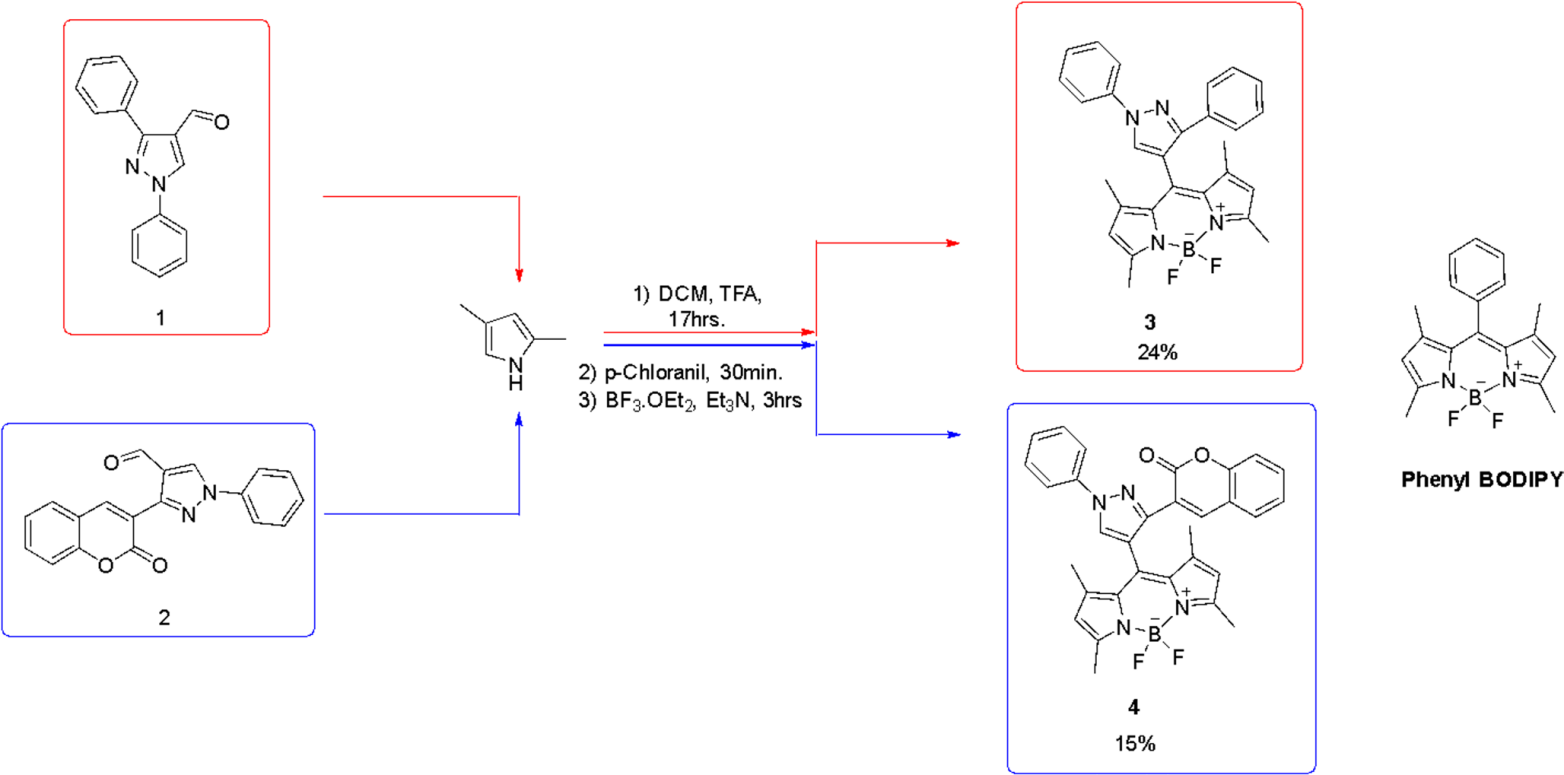
Reagents and synthesis route of the target compounds **3** and **4**, and the structure of phenyl BODIPY for comparison with **3** and **4**

In this study, phenyl- and coumarin-substituted pyrazole-4-carbaldehydes **1** and **2** were used as starting materials for the synthesis of the target BODIPY derivatives, **3** and **4**. These pyrazole-4-carbaldehydes were synthesized according to previously reported procedures [26, 27]. With pyrazole-4-carbaldehydes **1** and **2** in hand, preparation of the corresponding meso pyrazole-substituted BODIPYs was undertaken. The targeted compound **3** was synthesized by the acid-catalyzed reaction of diphenyl-pyrazole-4-carbaldehyde **1** and 2,4-dimethylpyrrole in trifluoroacetic acid followed by oxidation using *p*-chloranil and subsequent treatment with boron trifluoride etherate and trimethylamine. This reaction successfully generated **3** in 24% yield. Compound **3** was characterized by ^1^H and ^13^C-NMR spectra, and mass spectrometry data. The ^1^H NMR spectrum of **3** showed the disappearance of the aldehyde proton at around 10.00 ppm, indicating that the starting material **1** was completely converted to compound **3**. The spectrum also showed a singlet at 5.93 ppm corresponding to the pyrrolic CH protons, and methyl protons on the pyrrole rings were observed at 1.68 and 2.47 ppm. The ^13^C NMR spectrum further showed the characteristic methyl resonances at 13.66 and 14.04 ppm. The molecular weight of compound **3** was determined by HR-MS, which confirmed the expected molecular ion at *m/z* 466. Following the successful synthesis of meso-substituted phenyl pyrazole BODIPY **3**, the synthesis of a related coumarin containing BODIPY was investigated. For this purpose, coumarin-pyrazole-4-carbaldehyde **2** was reacted with 2,4-dimethylpyrrole under the same reaction conditions to afford the corresponding compound **4** in a 15% yield. The ^1^H and ^13^C NMR spectra of **4** were consistent with the proposed structure (Scheme 2).

**Fig. 1:**
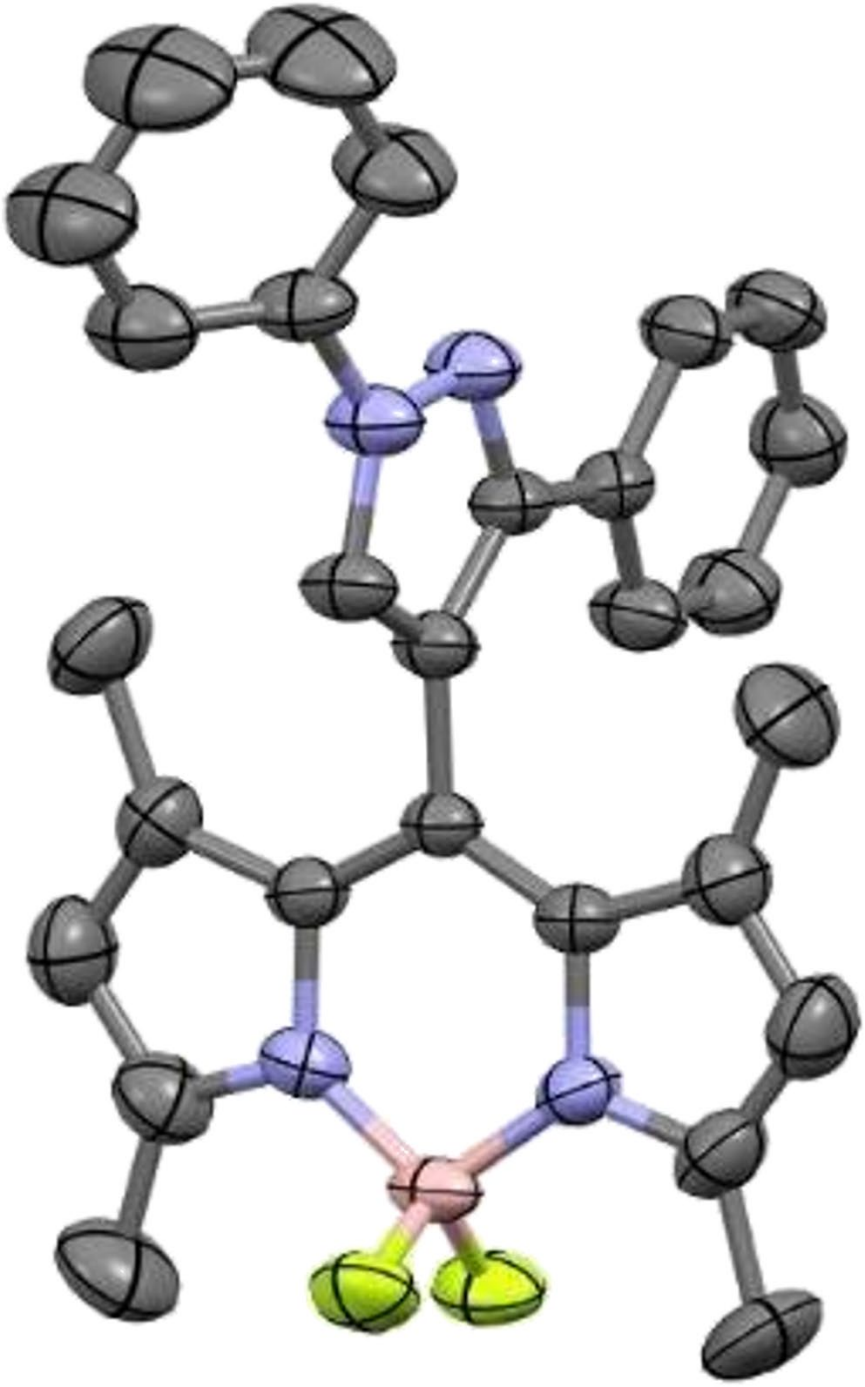
Crystal structure of compound **3**, showing displacement ellipsoids at the 50% probability level. Only one molecule of the asymmetric unit of compound **3** is shown

The single crystal structure of compound **3** was obtained by slow evaporation from DCM/MeOH. X-ray crystallographic analyses revealed that the compound crystallized in the triclinic *P-1* space group (Fig. 1, and Table S1). The F-B-F, N-B-N, and N-N-F bond angles, as well as the B-F bond lengths, were found to be consistent with the previously reported BODIPY units [39]. The dihedral angles between the meso-pyrazole group and the boron-dipyrromethene π-core were measured to be 79.35 ^ͦ^ for **3.**

### Photophysical Characterizations

**Fig. 2:**
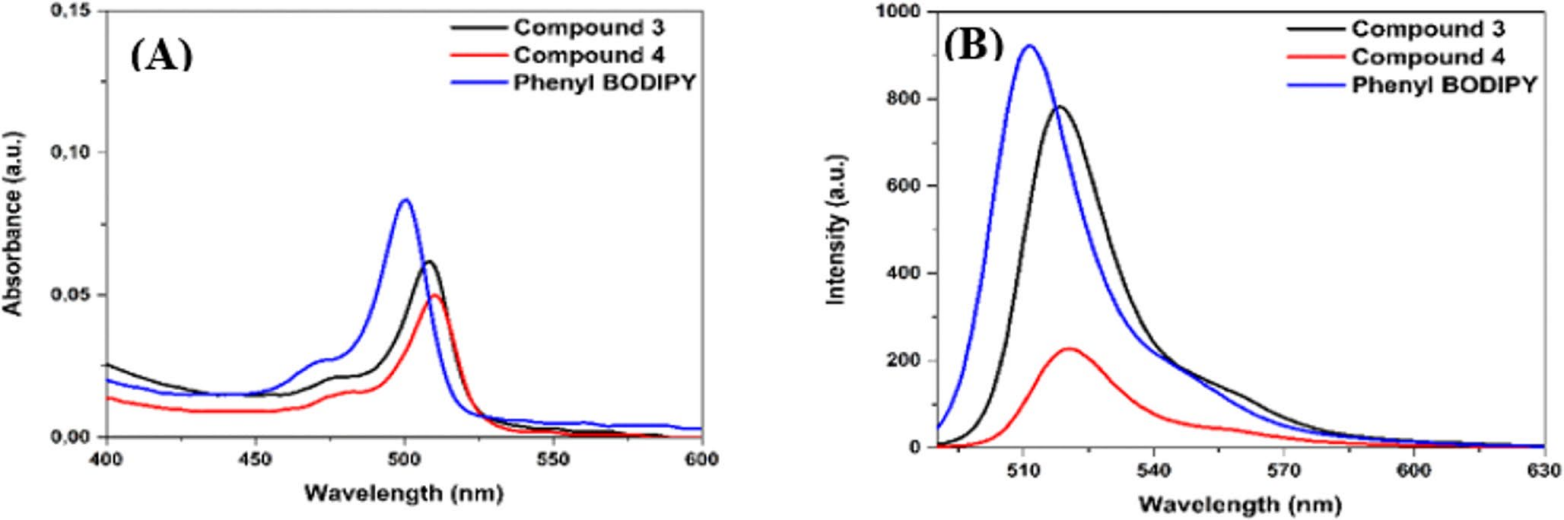
The absorbance (**A**) and fluorescence (**B**) spectra of 1 × 10^− 6^ M phenyl BODIPY, **3**, and **4** in DCM

**Fig. 3:**
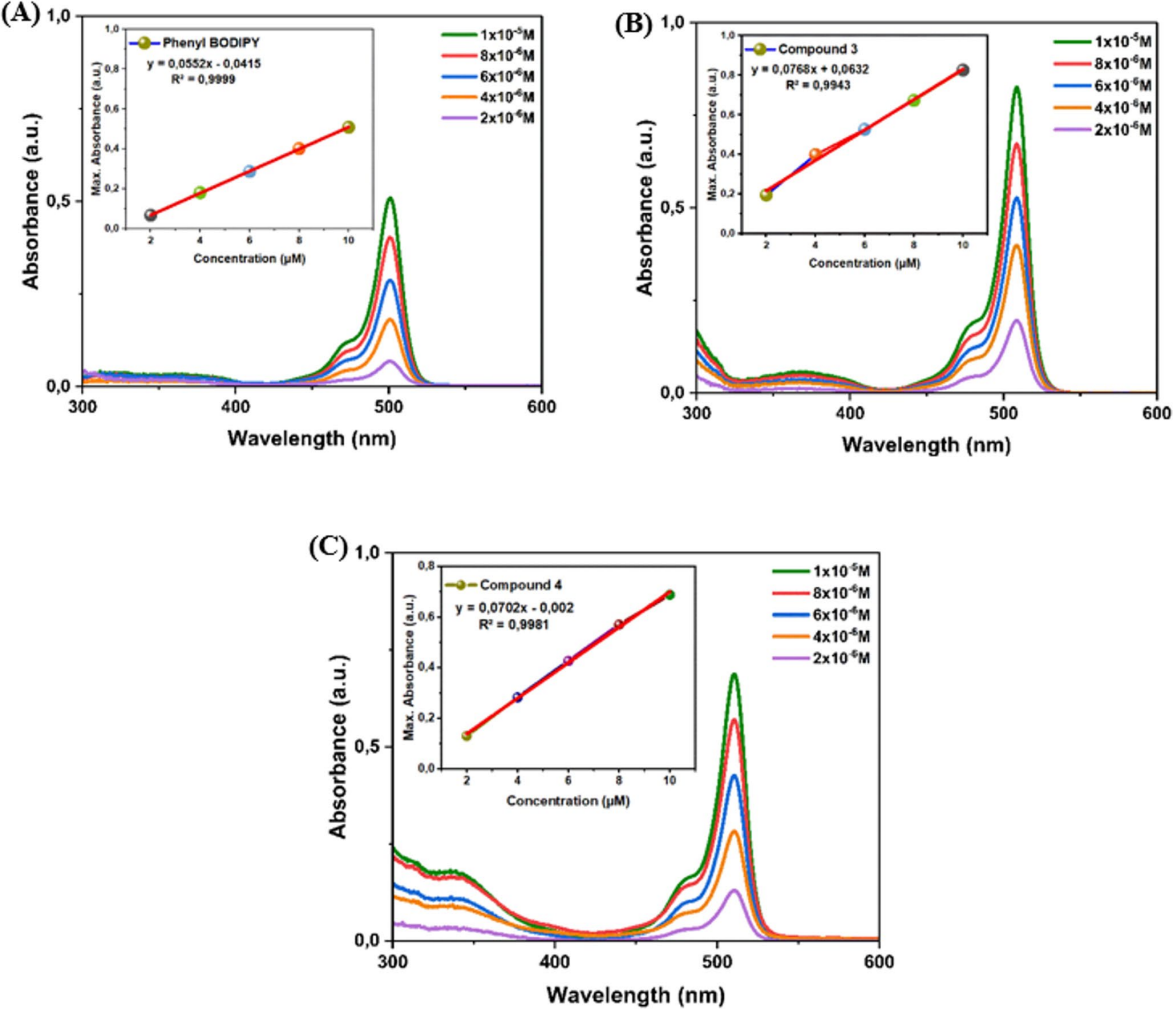
Absorption spectra of compounds (**A**) phenyl BODIPY, (**B**) **3**, and (**C**) **4** in DCM at different concentrations

**Fig. 4:**
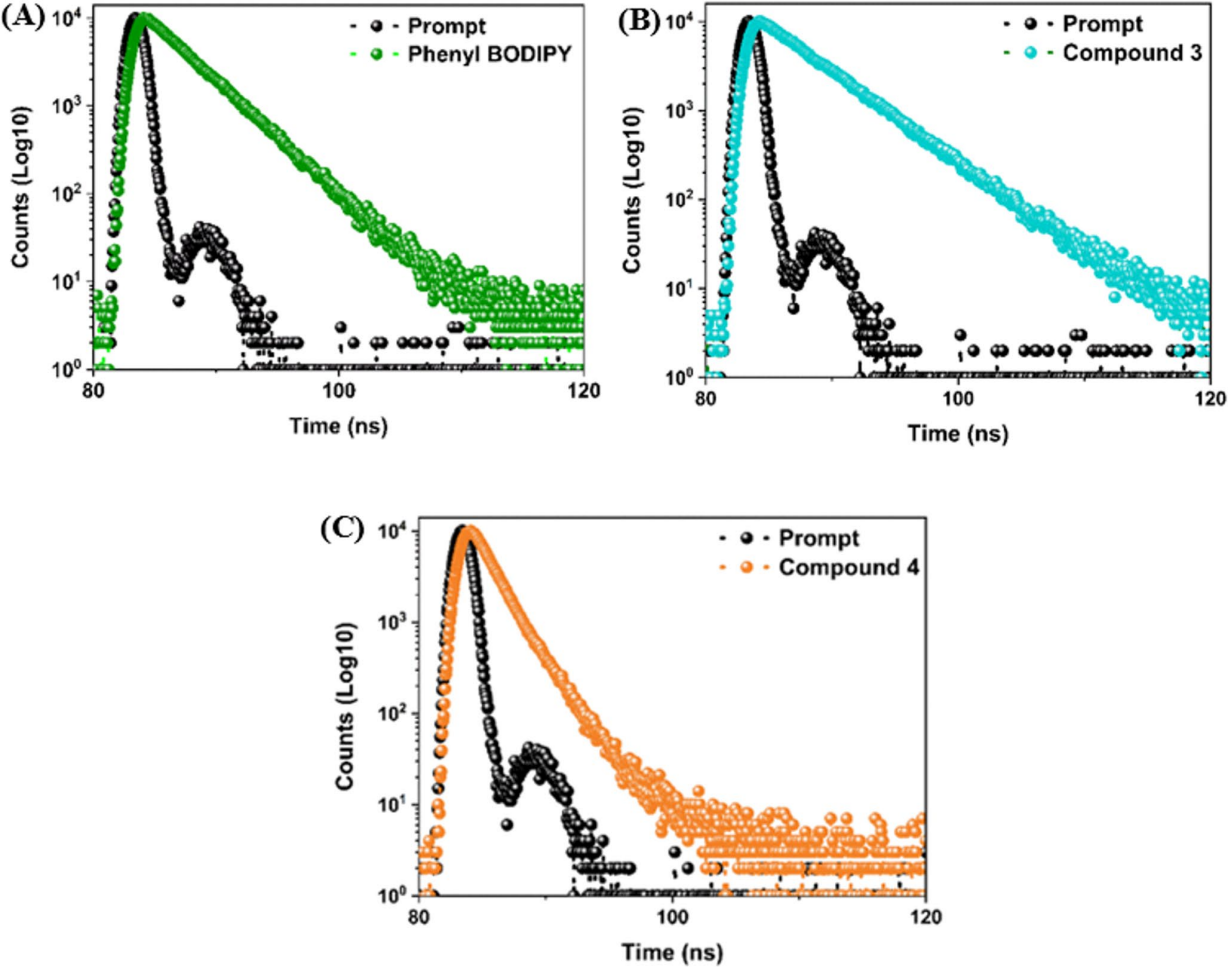
Fluorescence decay profile of (**A**) phenyl BODIPY, (**B**) **3**, and (**C**) **4** in DCM. In each graph, the black curve is the “prompt” signal, the reference pulse representing the response time of the laser pulse in the system. The colored curves show the fluorescence response of the corresponding compound after excitation

To investigate the photophysical properties of the newly synthesized compounds **3** and **4**, UV-Vis and time-resolved fluorescence spectroscopy were employed. To evaluate the environmental sensitivity of the synthesized dyes, the electronic absorption and fluorescence spectra of the compounds were measured in a range of solvents, including ethyl acetate, cyclohexane, toluene, 1,4-dioxane, chloroform, DCM, ethanol, THF, ACN, DMF, acetone, and DMSO (Figure S17-22). Both compounds **3** and **4** maintained their spectral profiles regardless of solvent polarity; the absorption and emission peaks remained nearly constant, with marginal shifts of only ± 3–5 nm. This photophysical stability indicates that the electronic transitions of these dye molecules are well shielded from external environmental changes. On the other hand, the observed decreases in fluorescence intensity, particularly in solvents such as ethanol, are attributed to specific interactions like hydrogen bonding between the solvent molecules and the dye. These interactions do not alter the electronic energy levels of the molecule, but rather cause some of the excited state energy to be dissipated through paths other than emission, thereby reducing the fluorescence intensity. Absorption and fluorescence measurements of **3** and **4** were performed at a concentration of 1 × 10^− 6^ M at room temperature with an excitation wavelength of 475 nm (Figs. 2). Accordingly, the compounds demonstrated absorption maxima (λ_abs_) at 508 nm (**3**) and 510 nm (**4**), corresponding to S_0_→S_1_ transitions. Their emission maxima (λ_em_) were observed at 518 nm and 520 nm for **3** and **4**, respectively, exhibiting a 10 nm Stokes shift, in agreement with the typical behavior of BODIPY dyes, which generally show small Stokes shifts [28], suggesting minimal reabsorption losses. As presented in Fig. 2B, compound **4** exhibited lower fluorescence intensity and lower fluorescence quantum yield compared to compound **3** and the phenyl-substituted analogue, indicating a relatively reduced emissive efficiency for compound **4.** The molar extinction coefficients (ε) were measured as 8.26 × 10^4^ M^−1^cm^− 1^ for **3** and 6.88 × 10^4^ M^− 1^ cm^− 1^ for **4** (Fig. 3 and Table 1), the intense absorption bands observed in the visible region together with the high molar absorption coefficients of compounds **3** and **4** indicate efficient photon absorption by the BODIPY chromophore. These features are characteristic of BODIPY-based systems and suggest that the synthesized compounds possess effective light-harvesting ability, which is an important property for fluorescence-related and photoactive applications [40, 41]. This reduced fluorescence intensity for **4** correlates with the fluorescence lifetime data (Fig. 4), as expected. The fluorescence lifetimes (τ) were 4.17 ns and 1.67 ns for the respective compounds, with **4** exhibiting a rapid fluorescence decay attributed to the coumarin-based structural modifications. These differences in terms of the structural features of compound 4, which may facilitate enhanced intramolecular motions and non-radiative decay pathways, resulting in reduced fluorescence lifetime and quantum yield. Therefore, the prepared compounds may be promising candidates for certain bioimaging applications, particularly those involving dynamic intracellular processes. Additionally, the phenyl and coumarin groups contributed distinct photophysical profiles. As seen in Table 1, the quantum yields of the phenyl BODIPY **3** and **4** were found to be 0.82 and 0.33, respectively.

**Table 1:** Photophysical properties of synthesized compounds

Compound^a^	λ_ab_(nm)	λ_em_(nm)	∆_Stokes_(nm)	є^b^ (10^4^ M^− 1^ cm^− 1^)	τ_F_^c^ (ns)	Quantum Yield
Phenyl BODIPY	501	511	10	3.40	3.30 CHISQ: 1,044	0.93
3	508	518		8.26	4.17 CHISQ: 1,177	0.82
4	510	520		6.88	1.67 CHISQ: 1,508	0.33

^a^ DCM, ^b^ Molar extinction coefficients, ^c^ Lifetime

The 3D spectrum of compound **3** exhibits a broad absorption band and intense emission signals (Figure S23-25). In contrast, **4** displays strong fluorescence across a wide absorption range, which may facilitate faster response dynamics and offer an advantage in rapid intracellular imaging applications. These distinctive photophysical properties highlight these BODIPY derivatives as versatile tools for fluorescence-based bioimaging.

### Biological Evaluations

**Fig. 5:**
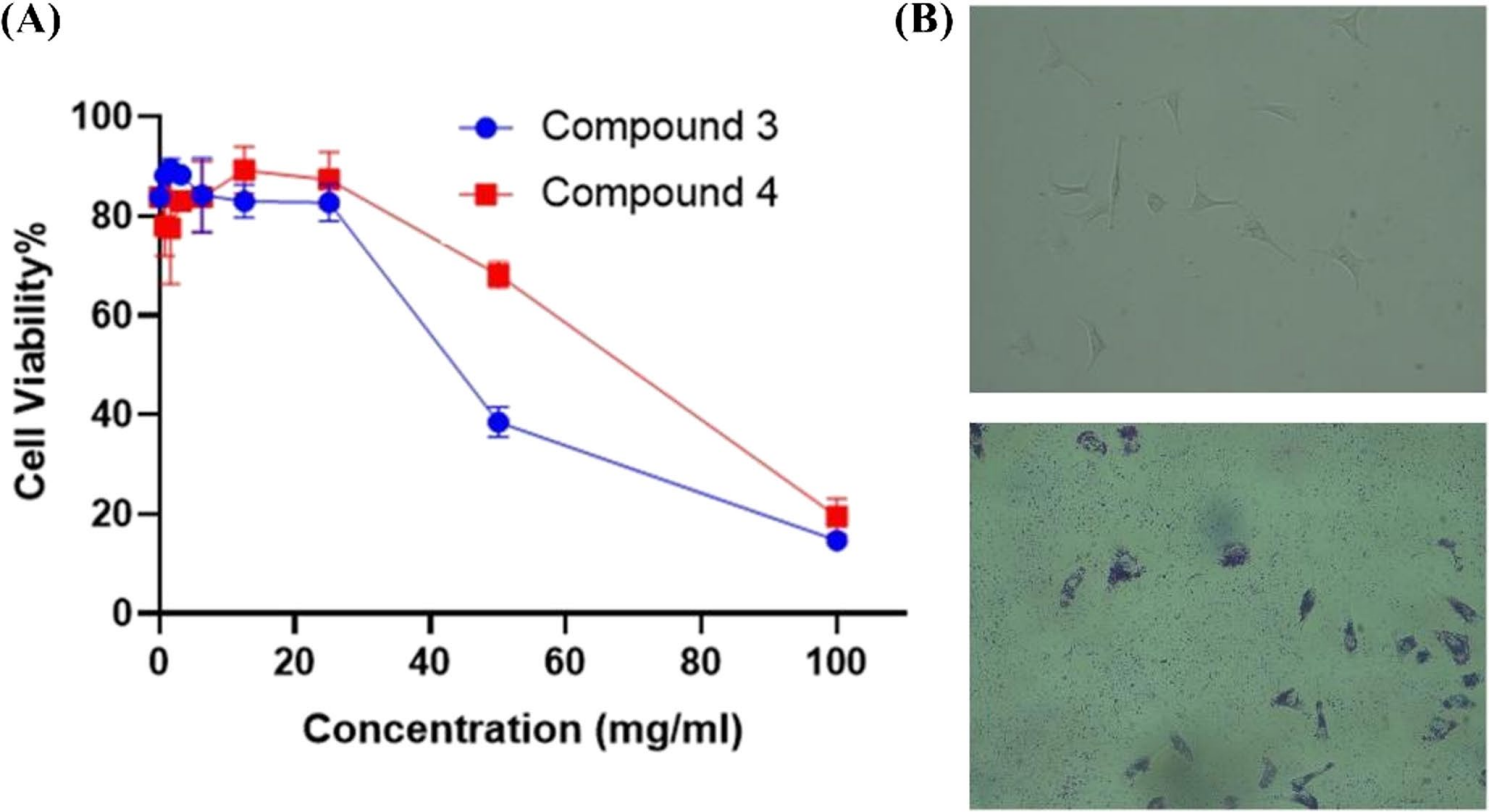
Effects of **3** and **4** on the viability of the HCT-116 cells. (**A**) MTT cell viability assay showing concentration-dependent toxicity of the compounds. (**B**) A representative image for Trypan Blue staining of compound (**3**)-treated cells, underlying disrupted cellular morphologies and blue-staining (bottom) compared to DMSO-treated cells (top)

Following successful synthesis and structural characterization, compounds **3** and **4** were evaluated for cytotoxicity and fluorescence imaging in the human colon cancer cell line HCT-116. Cytotoxicity assays revealed that both compounds exhibited cytotoxicity at high concentrations (Fig. 5A). The IC50 values were 47.46 ± 3.69 and 83.56 ± 2.40 mg/ml for **3** and **4**, respectively. Cytotoxicities of the compounds were further proved by Trypan Blue treatment, staining the death cells to blue and underlying necrotic or apoptotic cell death mechanisms (Fig. 5B). However, this approach could not distinguish apoptotic versus necrotic cell death but only verified the viability results. Although BODIPY itself is generally considered non-toxic, pyrazole-4-carbaldehyde derivatives have been reported to exhibit toxicity against various organisms and cancer cells [42–44]. At the molecular level, the toxic effects of pyrazole-containing compounds have been attributed to alterations in intracellular ion balances [43]. Furthermore, modifying BODIPY core with pyrazole-containing groups has been reported to induce cytotoxicity against diverse cancer cells at high concentrations, supporting the present study [19–24]. Notably, compound **4** contains a coumarin unit, a well-known toxic compound that has been extensively studied in cancer research [45]. Moreover, combining coumarin and pyrazole units has been shown to enhance cellular toxicity [46–48]. However, in this study, the toxicity of the coumarin-containing compound **4** did not show higher toxicity than compound **3**, possibly due to limited cellular internalization and/or aggregation problems of **4**, requiring further molecular studies.

**Fig. 6:**
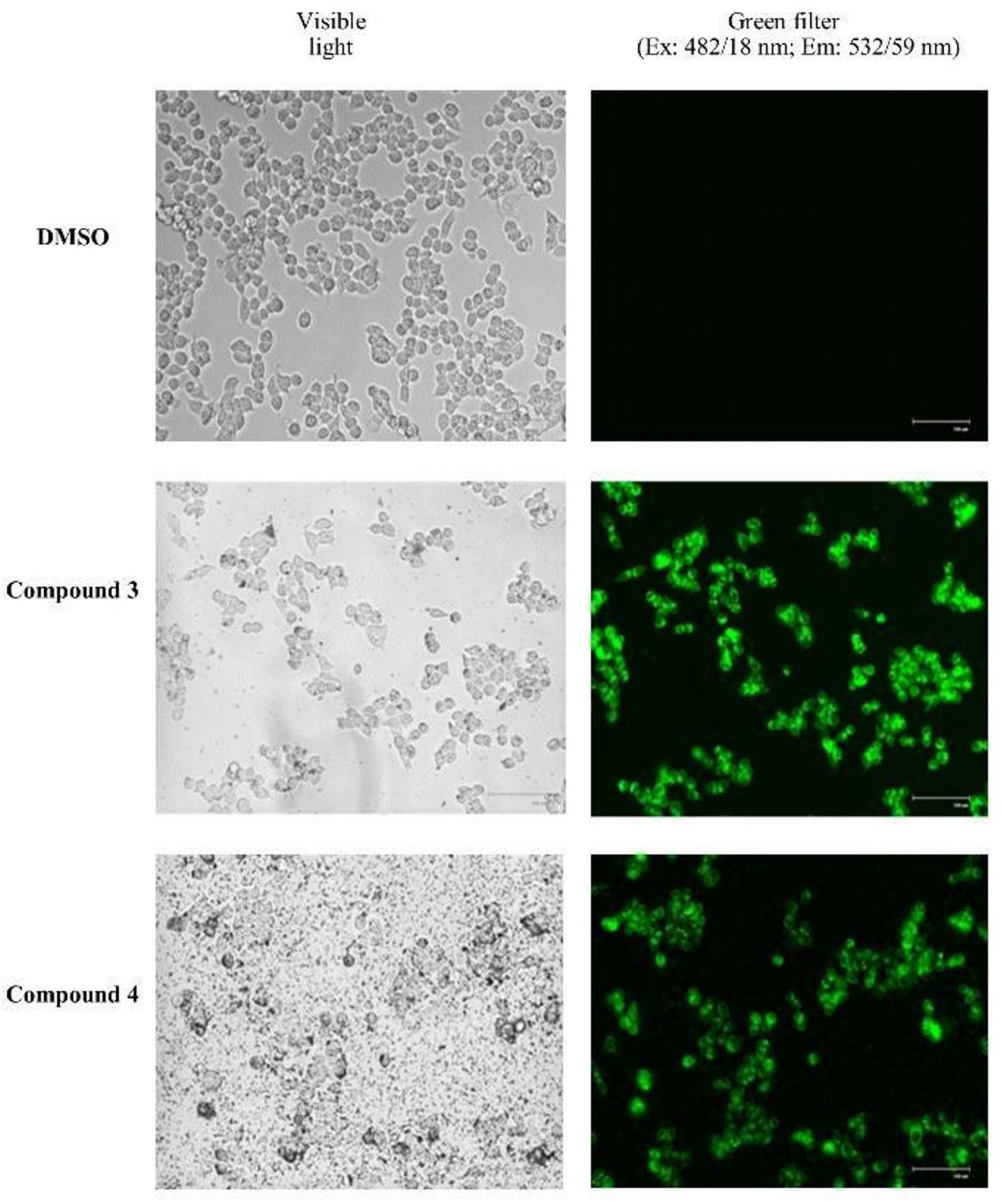
Live cell imaging and internalization patterns of **3** and **4**

Live-cell imaging using a fluorescence microscope was performed to investigate the internalization of the compounds by the cancer cells. Trypan Blue was used to quench extracellular signal and obtain only intracellular fluorescence [49]. Although sub-cellular localizations could not be determined due to microscope limitations, the results indicated that the compounds were internalized by the cells, as confirmed using a filter with an excitation wavelength of 482/18 nm and an emission wavelength of 532/59 nm (Fig. 6). At the same concentrations (1 mg/ml), compound **4** showed lower fluorescence intensity than compound **3**, suggesting limited internalization. Furthermore, compound **4** displayed solubility and aggregation issues in biological media. Consequently, further investigation focused on potential alterations in the fluorescence properties of **4**.

**Fig. 7:**
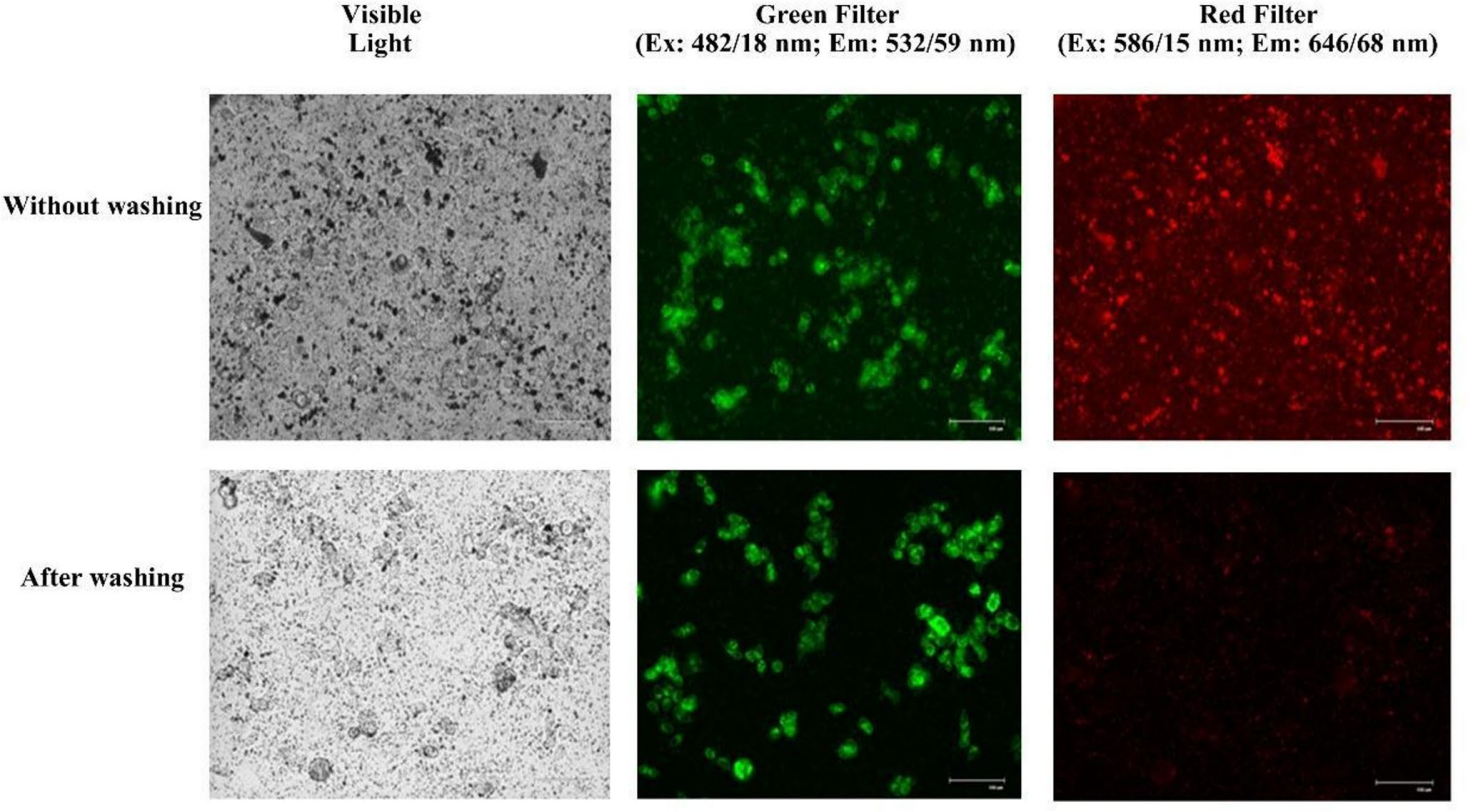
Live cell imaging by compound **4** before and after washing steps

To investigate fluorescence changes arising from solubility and aggregation issues, cells treatment with compound **4** were intentionally not washed and Trypan Blue was omitted. The results showed that the aggregation of **4** on cells, due to the absence of a washing step, resulted in secondary fluorescence, detected using a filter set with an excitation at 586/15 nm and an emission at 646/68 nm (Fig. 7). Aggregation-induced fluorescence changes have previously been reported for the coumarin-containing complexes, consistent with our observations [50–52]. Still, further studies focusing on concentration-dependent fluorescence alterations with and without culture medium optimizations are required to decipher the aggregation-dependent photophysical changes for **4**.

## Conclusion

In this study, two novel pyrazole-linked BODIPY compounds were successfully synthesized, characterized, and assessed for their photophysical and primary biological properties. Spectroscopic analysis demonstrated strong light absorption and efficient fluorescence emission, exhibiting favorable features such as high molar extinction coefficients and minimal Stokes shifts, highlighting their potential for bioimaging applications. Structural modifications incorporating phenyl and coumarin motifs, enabled distinct optical properties, enhancing their suitability for a range of imaging applications. Biological evaluation demonstrated significant fluorescence activity at specific concentrations, enabling efficient cellular imaging. Although cytotoxicity at higher doses (47.46 ± 3.69 mg/ml for **3** and 83.56 ± 2.40 mg/ml for **4**), the rapid fluorescence decay of the coumarin-substituted BODIPY (**4**) and the photophysical stability of the phenyl-substituted counterpart (**3**) highlight the versatility of BODIPY derivatives for targeted biomedical applications. Overall, this study emphasizes the significance of meso-functionalized pyrazole BODIPYs in expanding the utility of BODIPY dyes for biomedical imaging. These findings pave the way for the development of advanced fluorophores tailored to specific bioimaging needs as well as other optical applications, including sensing and light-harvesting technologies.

## Supplementary Information

Supplementary data was given as Supporting Information.

Supplementary Material 1

## Data Availability

No datasets were generated or analysed during the current study.
