# Supplementary material for: Synthesis, Photophysical Properties, and Primary Biological Characteristics of Meso-pyrazole-substituted BODIPY Derivatives
Source: J Fluoresc. 2026 Jun 24;36(7):4347–60. doi: 10.1007/s10895-026-04852-y (PMC13486093; doi:10.1007/s10895-026-04852-y)
Supplement: Supplementary file 1 [file 10895_2026_4852_MOESM1_ESM.docx]

**SUPPORTING INFORMATION**

**Synthesis, Photophysical Properties, and Primary Biological Characteristics of Meso-pyrazole-substituted BODIPY Derivatives**

Zehra COŞKUN^a^, Ercan COŞAR^a^, Burcu TOPALOĞLU AKSOY ^a^, Hasan Hüseyin KAZAN^b^, Ibrahim F. SENGUL^a^*, Bünyemin ÇOŞUT^a*^

^a^ Department of Chemistry, Gebze Technical University, 41400, Gebze, Türkiye

^b^ Department of Medical Biology, Gulhane Faculty of Medicine, University of Health Sciences, Ankara, Türkiye

*Correspondences: [fazilsengul@gtu.edu.tr](mailto:fazilsengul@gtu.edu.tr), [bcosut@gtu.edu.tr](mailto:bcosut@gtu.edu.tr)

**TABLE OF CONTENTS**

**Figure S1. ^1^**H NMR Spectrum of Compound **1**	3

**Figure S2. ^13^**C NMR Spectrum of Compound **1**	3

**Figure S3. ^1^**H NMR Spectrum of Compound **2**	4

**Figure S4. ^13^**C NMR Spectrum of Compound **2**	4

**Figure S5.** HR-MS Spectrum of Compound **3**	5

**Figure S6. ^1^**H NMR Spectrum of Compound **3**	5

**Figure S7. ^13^**C NMR Spectrum of Compound **3**	6

**Figure S8.** ^11^B NMR spectrum of Compound **3**	6

**Figure S9.** ^19^F NMR Spectrum of Compound **3**	7

**Figure S10.** FT-IR Spectrum of Compound **3**	7

**Figure S11.** HR-MS Spectrum of Compound **4**	8

**Figure S12.** ^1^H NMR Spectrum of Compound **4**	8

**Figure S13. ^13^**C NMR Spectrum of Compound **4**	9

**Figure S14.** ^11^B NMR spectrum of Compound **4**	9

**Figure S15.** ^19^F NMR spectrum of Compound **4**	10

**Figure S16.** FT-IR Spectrum of Compound **4**	10

**Figure S17.** Absorbance Spectrum of Phenyl BODIPY in Different Solvents	11

**Figure S18.** Fluorescence Spectrum of Phenyl BODIPY in Different Solvents	11

**Figure S19.** Absorbance Spectrum of Compound **3** in Different Solvents	12

**Figure S20.** Fluorescence Spectrum of Compound **3** in Different Solvents	12

**Figure S21.** Absorbance Spectrum of Compound **4** in Different Solvents	13

**Figure S22.** Fluorescence Spectrum of Compound **4** in Different Solvents	13

**Figure S23.** 3D Spectrum of Phenyl BODIPY	14

**Figure S24.** 3D Spectrum of Compound **3**	14

**Figure S25.** 3D Spectrum of Compound **4**	15

**Table S1:** Crystal data and refinement parameters for compound 3	16

**Figure S1.** **^1^**H NMR Spectrum of Compound **1**

**Figure S2**. ^13^C NMR Spectrum of Compound 1

**Figure S4. ^13^**C NMR Spectrum of Compound **2**

**Figure S3.** ^1^H NMR Spectrum of Compound 2

**Figure S SEQ Figure \* ARABIC 4.** ^13^C NMR Spectrum of Compound 2

**Figure S5.** HR-MS Spectrum of Compound **3**

**Figure S6. ^1^**H NMR Spectrum of Compound **3**

**Figure S7. ^13^**C NMR Spectrum of Compound **3**

**Figure S8.** ^11^B NMR spectrum of Compound **3**

**Figure S9.** ^19^F NMR spectrum of Compound **3**

**Figure S10.** FT-IR Spectrum of Compound **3**

**Figure S11.** HR-MS Spectrum of Compound **4**

**Figure S12. ^1^**H NMR Spectrum of Compound **4**

**Figure S13. ^13^**C NMR Spectrum of Compound **4**

**Figure S14.** ^11^B NMR spectrum of Compound **4**

**Figure S15.** ^19^F NMR spectrum of Compound **4**

**Figure S16.** FT-IR Spectrum of Compound **4**

**Figure S17.** Absorbance Spectrum of Phenyl BODIPY in Different Solvents

**Figure S18.** Fluorescence Spectrum of Phenyl BODIPY in Different Solvents

**Figure S19.** Absorbance Spectrum of Compound **3** in Different Solvents

**Figure S20.** Fluorescence Spectrum of Compound **3** in Different Solvents

**Figure S21.** Absorbance Spectrum of Compound **4** in Different Solvents

**Figure S22.** Fluorescence Spectrum of Compound **4** in Different Solvents

**Figure S23.** 3D Spectrum of Phenyl BODIPY

**Figure S24.** 3D Spectrum of Compound **3**

**Figure S25.** 3D Spectrum of Compound **4**

**Table S4.** Crystal data and refinement parameters for compound **3.**

| Empirical Formula | C_28_H_25_BF_2_N_4_ |
| --- | --- |
| Formula weight/g. mol^-1^ | 466.33 |
| Temperature/K | 273.15 |
| Wavelength (Å) | MoKα (λ = 0.71073) |
| Crystal System | Triclinic |
| Space group | P-1 |
| a/Å | 10.677(2) |
| b/Å | 11.389(2) |
| c/Å | 20.618(4) |
| α/° | 94.633(3) |
| β/° | 94.976(4) |
| γ/° | 99.741(3) |
| Crystal size/mm^3^ | 0.237 × 0.19 × 0.11 |
| V/ Å^3^ | 2450.0(9) |
| Z | 4 |
| ρcalcd (g. cm^−3^) | 1.264 |
| µ (mm^−1^) | 0.086 |
| F(000) | 976.0 |
| θ range for data collection/° | 3.646 to 54.968 |
| h/k/l | -13 ≤ h ≤ 13, -14 ≤ k ≤ 14, -26 ≤ l ≤ 26 |
| Reflections collected | 39991 |
| Independent reflections | 11238 [R_int_ = 0.0885 |
| Data/restraints/parameters | 11238/0/639 |
| Goodness-of-fit on F^2^ (S) | 0.926 |
| Final R indices [I > 2σ(I)] | R_1_ = 0.0628, wR_2_ = 0.1581 |
| R indices (all data) | R_1_ = 0.1488, wR_2_ = 0.2041 |
| Largest diff. peak/hole / e Å^-3^ | 0.18/-0.27 |
